# The role of livestock movements in the spread of Rift Valley fever virus in animals and humans in Mayotte, 2018–19

**DOI:** 10.1371/journal.pntd.0009202

**Published:** 2021-03-08

**Authors:** Younjung Kim, Raphaëlle Métras, Laure Dommergues, Chouanibou Youssouffi, Soihibou Combo, Gilles Le Godais, Dirk U. Pfeiffer, Catherine Cêtre-Sossah, Eric Cardinale, Laurent Filleul, Hassani Youssouf, Marion Subiros, Guillaume Fournié

**Affiliations:** 1 Department of Infectious Diseases and Public Health, Jockey Club College of Veterinary Medicine and Life Sciences, City University of Hong Kong, Hong Kong, China; 2 INSERM, Sorbonne Université, Institut Pierre Louis d’Épidémiologie et de Santé Publique (UMRS-1136), Paris, France; 3 La Coopération Agricole, Paris, France; 4 GDS 976 Mayotte, Combani, France; 5 Direction de l’Alimentation, de l’Agriculture et de la Forêt de Mayotte, Mamoudzou, France; 6 Veterinary Epidemiology, Economics and Public Health group, Department of Pathobiology and Population Sciences, The Royal Veterinary College, Hatfield, United Kingdom; 7 CIRAD, UMR ASTRE, Sainte Clotilde, La Réunion, France; 8 ASTRE, CIRAD, Univ Montpellier, INRAE, Montpellier, France; 9 Santé Publique France, Mamoudzou, France; Oregon State University College of Veterinary Medicine, UNITED STATES

## Abstract

Rift Valley fever (RVF) is a vector-borne viral disease of major animal and public health importance. In 2018–19, it caused an epidemic in both livestock and human populations of the island of Mayotte. Using Bayesian modelling approaches, we assessed the spatio-temporal pattern of RVF virus (RVFV) infection in livestock and human populations across the island, and factors shaping it. First, we assessed if (i) livestock movements, (ii) spatial proximity from communes with infected animals, and (iii) livestock density were associated with the temporal sequence of RVFV introduction into Mayotte communes’ livestock populations. Second, we assessed whether the rate of human infection was associated with (a) spatial proximity from and (b) livestock density of communes with infected animals. Our analyses showed that the temporal sequence of RVFV introduction into communes’ livestock populations was associated with livestock movements and spatial proximity from communes with infected animals, with livestock movements being associated with the best model fit. Moreover, the pattern of human cases was associated with their spatial proximity from communes with infected animals, with the risk of human infection sharply increasing if livestock in the same or close communes were infected. This study highlights the importance of understanding livestock movement networks in informing the design of risk-based RVF surveillance programs.

## Author summary

Rift Valley fever (RVF) is a vector-borne zoonotic disease, endemic in many sub-Saharan Africa regions with substantial outbreaks. RVF virus (RVFV) is transmitted to animals primarily by the bite of infected mosquitos, whereas direct or indirect contact with infected animals forms the primary route of RVFV transmission to humans. In 2018–19, Mayotte, an archipelago in the Indian Ocean between Madagascar and the coast of Eastern Africa, experienced an RVF epidemic in both livestock and humans. In this study, we investigated factors shaping the spatio-temporal pattern of RVFV infection in livestock and human populations across Mayotte. The diffusion of RVFV through Mayotte’s livestock population was associated with livestock movements and, potentially to a lesser extent, spatial proximity from communes with infected animals. Moreover, the pressure of infection on humans was the highest if nearby livestock were infected. This study highlights the value of accounting for the structure of livestock movement networks in the surveillance of zoonotic diseases at the human-animal interface, and the need for One Health approaches.

## Introduction

Livestock movements are a major pathway for the spread of many infectious diseases, including those with zoonotic potential [1–3]. The structure of livestock movement networks has been analyzed to identify epidemiological units (e.g. farms and geographical areas) at high risk of becoming infected and spreading infection, and to inform the design of risk-based surveillance programs [4,5]. Targeting surveillance efforts towards those units would be expected to allow the early detection of infectious disease outbreaks, promoting their effective management [6].

**Fig 1:**
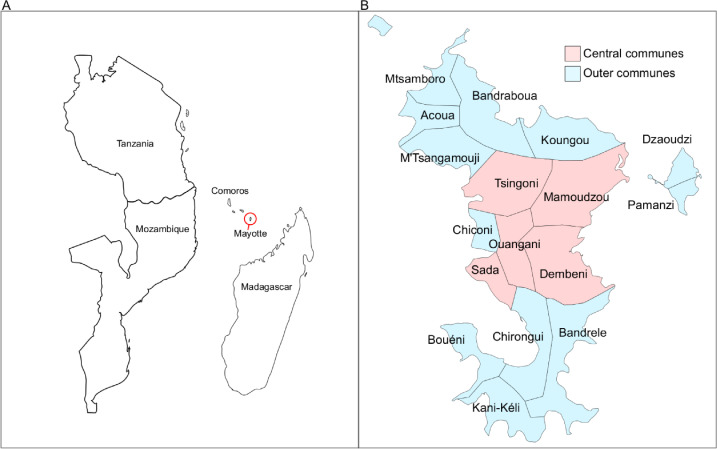
**Geographical location of Mayotte (A) and its communes (B).** In Fig 1B, communes were classified into central (red) or outer (blue) communes based on their structural equivalence in the livestock movement network [11].

However, compared to other livestock infectious diseases, our understanding of the role of livestock movements in the spread of Rift Valley fever (RVF) is limited. RVF is a vector-borne and zoonotic viral disease of major animal and public health importance. RVF virus (RVFV), a member of the genus *Phlebovirus* in the family *Phenuiviridae*, infects animals primarily by the bite of infected mosquitos, whereas contact with infected animal blood, body fluids, or tissues forms the major route of RVFV transmission to humans [7]. RVFV is endemic in many sub-Saharan Africa regions and has been likely introduced into the island of Mayotte, a French department in the Mozambique Channel of the Indian Ocean, through the movements of people and animals from the Union of the Comoros [8] (Fig 1A). In the last ten years, Mayotte has experienced two RVF epidemics, in 2008–10 [9] and 2018–19 [10]. In the 2008–10 epidemic, RVFV antibody prevalence patterns suggested that the network of livestock movements influenced the spatial dissemination of the virus among the island’s livestock population [11]. Among the island’s 17 communes (i.e. administrative regions), in the early stage of the 2008–10 epidemic, seroprevalence was significantly higher in *central* communes, which formed a group of communes densely connected via livestock movements, than in *outer* communes, which were weakly connected with one another and received most livestock from central communes. Antibody prevalence in outer communes subsequently increased to the level experienced in central communes [11]. Livestock movements were not, however, explicitly modelled, and their potential contribution to RVFV dissemination was not quantified [11].

Several RVFV transmission models have provided useful insights into the contribution of different host species, vector and environmental factors to RVFV spread [12–17]. While some models used proxy measures (e.g. distance and livestock populations) to capture animal movement patterns [14,15], others were informed by animal movement survey data focusing on the role of cattle movements in maintaining viral circulation in RVFV-endemic livestock populations [16,17]. However, none explored the role of livestock movements in RVFV dissemination during an epidemic following its incursion into a susceptible population by combining both animal movement and epidemic case data. In particular, RVFV transmission models focusing on Mayotte have not considered the movements of livestock on the island. Additionally, although around half of human infections were estimated to have resulted from direct contact with infected animals during this 2018–19 epidemic [18], no studies have analyzed, so far, the potential implications of livestock movements for human infections based on both animal and human epidemic case data.

In November 2018, RVFV re-emerged in Mayotte. Between November 2018 and August 2019, a total of 165 animals and 143 humans tested positive for RVFV [10,19]. Outbreaks in livestock affected 16 out of the island’s 17 communes, and human cases were reported in all communes. Such concurrent epidemics in livestock and humans provided a unique opportunity to explore the role of livestock movements in the 2018–19 RVF epidemic in both animals and humans, and the possible implications for risk-based surveillance and control programs. This study had two objectives. The first objective was to assess whether (i) livestock movements, (ii) spatial proximity from communes with infected animals, and (iii) livestock density were associated with the temporal sequence of RVFV introduction into communes’ livestock populations. The second objective was to assess whether the rate of human infection was associated with (a) spatial proximity from and (b) livestock density of communes with infected animals.

## Methods

### Data

Livestock and human case data for the 2018–19 Mayotte RVF epidemic were provided by the Veterinary Services of Mayotte, the CoopADEM (Coopérative des Eleveurs de Mayotte), and Santé Publique France. Livestock referred to cattle, sheep, and goats. Farmers were sensitized to the disease and encouraged to report clinical signs suggestive of RVFV infection (e.g. abortions or pyrexia) to the animal disease surveillance system (SESAM, Système d’épidémiosurveillance animale à Mayotte). Suspected livestock identified through this passive surveillance system were tested for RVFV by RT-PCR [20], and those that tested positive were defined as livestock cases of RVF. The proportion of farms with a declared veterinarian was only slightly higher in central (30.7%) than outer communes (26.6%) [21]. Also, due to the small size of the island (374km^2^), all farms in Mayotte were located less than one hour away, by car, from the nearest private veterinary practice (Dommergues, personal communication). This suggests that the coverage of veterinary services, and the level of underreporting were likely to be similar across the island. Human cases of RVF were individuals who tested positive by RT-PCR [20] following a consultation with a general practitioner for a dengue-like syndrome. For each case, animal or human, the sampling date and the commune where the farm was located, or where the person was a resident, were recorded. While samples were collected from humans on the day of their medical consultation, samples were collected from animals within two weeks following the onset of clinical signs suggestive of an RVFV infection (Dommergues, personal communication).

We used livestock movement records for the period of 2007–14, since such data were not available for 2018–19. A previous analysis of these data showed that the yearly networks were strongly correlated with one another, suggesting that the overall structure of the Mayotte livestock movement network was stable during that period [11]. Also, while the size of livestock populations in each commune likely varied over the past decade, the ranking of those communes according to their number of livestock was similar between the 2010 agricultural census [22] and a survey conducted in 2015 [23] (p = 0.015, Spearman’s rank correlation *ρ* = 0.61). Moreover, there was no livestock movement restriction during the epidemic. Therefore, assuming that the network structure remained stable over the following six years including the 2018–2019 epidemic, we computed the average daily number of livestock movements between communes in 2007–2014 and used it as an estimation of livestock movement patterns in 2018–2019. We also classified communes into one of two clusters (i.e. central or outer), based on their level of structural equivalence [24] (Fig 1B). Briefly, two communes were considered structurally equivalent if they received and sent the same number of animals from, and to, the same communes. We described the temporal changes in the number of livestock cases in each cluster. The size of the livestock population per commune was provided by the latest agricultural census conducted in 2010 [22], and for any two communes, the Euclidean distance between their geometric centroids was used as their geographical distance. There were on average 1,751 livestock per commune (IQR: 1,019–1,876, Fig A in S1 Text), and the average livestock density was 87.4/km^2^ per commune (IQR: 41.8–202.3/km^2^), according to the 2010 agricultural census. Finally, we assessed variations in Normalized Difference Vegetation Index (NDVI) across the island, during the study period as a proxy of mosquito abundance since it reflects the level of vegetation and presence of water (i.e. conditions promoting mosquito proliferation) [25].

### Models

#### Role of the Mayotte livestock movement network in the livestock epidemic

We hypothesized that the structure of the Mayotte livestock movement network was associated with the temporal sequence of RVFV introduction into communes’ livestock populations. Yet, this temporal sequence could have also been influenced by the spatial proximity between communes, or the density of communes’ livestock populations. To test these hypotheses, we fitted 20 models simulating RVFV spread among communes’ livestock populations to the livestock case data. Under the baseline model formulations, RVFV spread among communes was influenced by (i) livestock movements (‘livestock movement model for livestock infection’, Model L.1), (ii) spatial proximity from infected communes (‘spatial proximity model for livestock infection’, Model L.2), (iii) livestock density in communes (‘livestock density model for livestock infection’, Model L.3), or (iv) none of those factors (‘null model’ with a constant background transmission rate, Model L.4). Model L.1 was further divided into the weighted (Model L.1.1) or unweighted (Model L.1.2) model, depending on whether it accounted for the number of animals moved between any two communes or not (4 + 1 = 5 models). Each of these 5 models was fitted with or without considering a delay between the viral incursion in a commune and viral transmission among its livestock (5 × 2 = 10 models), and each of these 10 models was further evaluated assuming time-independent or time-dependent daily rates of infection (10 × 2 = 20 models), as described below.

In the livestock movement (Models L.1.1 and L.1.2) and spatial proximity (Model L.2) models, the overall rate of infection (*λ*_*i*,*d*_) exerted on the livestock population in commune *i* on day *d* was modelled as:

$$\lambda_{i,d}=p_{i,d}+q$$ (1)

*p*_*i*,*d*_ was the rate of infection resulting from commune *i*’s relationship with communes whose livestock populations were infected and infectious (‘livestock-infected communes’). In other words, it was the rate of infection resulting from introducing animals from livestock-infected communes (Models L.1.1 and L.1.2) or sharing borders with livestock-infected communes (Model L.2). In both models, *q* was the background rate of infection, which accounted for other transmission routes (e.g. additional mosquito dissemination). We assumed that all communes were exposed to the same background infection rate, as suggested by NDVI spatiotemporal patterns (Fig B in S1 Text).

In Models L.1.1 and L.1.2, *p*_*i*,*d*_ was expressed as:

$$p_{i,d}=\psi \sum_{j=1,j\neq i}\left(\mu_{i,j}I_{j,d}\right)$$ (2A)

*ψ* was the daily rate at which a commune became infected through the introduction of an animal moved from a livestock-infected commune. *μ*_*i*,*j*_ was informed by the 2007–14 livestock movement data and expressed in two different ways. In the weighted model (Model L.1.1), *μ*_*i*,*j*_ was the average daily number of animals moved from commune *j* to commune *i*, while in the unweighted model (Model L.1.2), it was the presence of animal movements between communes (i.e. if animals were moved from commune *j* to commune *i*, *μ*_*i*,*j*_ = 1). I_*j*,*d*_ was a variable indicating the infection status of commune *j* on day *d* (i.e. 1 if infected, otherwise 0).

In Model L.2, *p*_*i*,*d*_ was expressed as:

$$p_{i,d}=\sigma \sum_{j=1,j\neq i}\left(B_{i,j}I_{j,d}\right)$$ (2B)

*σ* was the daily rate at which communes sharing a border with livestock-infected communes became infected. B_*i*,*j*_ was a variable indicating whether communes *i* and *j* shared a border (1 if shared, 0 otherwise). We considered that the two communes on the neighboring island, Petite-Terre, shared a border with the nearest commune on the main island, Grande-Terre, assuming that high levels of human movements between those communes [26] were associated with the movements of animals.

In Model L.3, *λ*_*i*,*d*_ was assumed to increase with livestock density in commune *i* (i.e. the average number of animals per km^2^) (*L*_*i*_) to the power of ω:

$$\lambda_{i,d}=q{L_{i}}^{\omega }$$ (2C)

Finally, in the null model (Model L.4), *λ*_*i*,*d*_ = *q*.

Under the baseline model formulations, a commune was assumed to have become infected and infectious on the day its first livestock case was sampled, and daily rates of infection were fixed during the entire epidemic (‘time-independent models’). Alternative model formulations were also considered in order to account for a possible lag between the introduction of RVFV into a commune and its transmission to susceptible animals (‘time-lag models’), and temporal variations in infection rates due to changes in mosquito abundance (‘time-dependent models’). For the time-lag models, we assumed that a commune became infected 5 weeks and infectious 2 weeks before the day its first livestock case was sampled. This was to account for the time required for viral amplification in the vector population (2 weeks) [27,28], the maximum latent period in livestock (1 week) [29], and the estimated maximum delay between the onset of clinical signs and sample collection in livestock cases (2 weeks). For the time-dependent models, the study period was divided into 30-day periods, and the daily rates of infection were allowed to vary at each period *t*. We assumed that the changes in mosquito abundance impacted all transmission routes similarly. Therefore, in the time-dependent versions of the livestock movement (Models L.1.1 and L.1.2) and spatial proximity (Model L.2) models, the background rate of infection, *q*_*t*_, was expressed as *q*_*t*_, = *sψ*_*t*_ and *q*_*t*_, = *sσ*_*t*_, respectively, with *s* being a scaling parameter. The model parameters and variables are summarized in Table 1.

**Table 1:** Parameters and variables in the models investigating factors associated with the epidemic in livestock.

Model	Parameter or variable		
	Notation	Description	Prior
Models L.1–4	*λ*_*i*,*d*_	Overall daily rate of infection exerted on the livestock population in commune *i* on day *d*	NA
Models L.1–4 (time-independent)	*q*	Background rate of infection	Uniform (0, 100)
Models L.1–2	*p*_*i*,*d*_	Daily rate of infection resulting from commune *i*’s relationship with livestock-infected communes	NA
	*s*	Scaling parameter for time-dependent models	Uniform (0, 100)
Livestock movement (Models L.1.1 and L.1.2)	*ψ*	Daily rate at which a commune became infected through introduction of an animal from a livestock-infected commune	Uniform (0, 100)
	*μ*_*i*,*j*_	Average daily number (weighted model) or occurrence (unweighted model) of animals moved from commune *j* to commune *i*	NA
	I_*i*,*d*_	Indicator variable for infection status of commune *i* on day *d*	NA
Spatial proximity (Model L.2)	*σ*	Daily rate at which a commune sharing a border with livestock-infected communes became infected	Uniform (0, 100)
	B_*i*,*j*_	Indicator variable for border sharing between communes *i* and *j*	NA
	I_*i*,*d*_	Indicator variable for infection status of commune *i* on day *d*	NA
Livestock density (Model L.3)	*L*_*i*_	Average number of animals per km^2^ in commune *i*	NA
	ω	Exponent for livestock density	Uniform (0, 100)

NA = not applicable

For all models, the likelihood (*l*) of the observed temporal sequence of infection of Mayotte communes was expressed as follows:

$$l_{1_{i}}=\left(e^{-\sum_{d=1}^{d=t_{i}-1}\lambda_{i,d}}\right)\left(1-e^{-\lambda_{i,t_{i}}}\right)$$ (3A)

$$l_{2_{j}}=e^{-\sum_{d=1}^{d=t_{last}}\lambda_{j,d}}$$ (3B)

$$l=\prod_{i}l_{1_{i}}\prod_{j}l_{2_{j}}$$ (3C)

*t*_*i*_ was the day commune *i* became infected (i.e., report of its first livestock case), and *t*_*last*_ was the day after the last reported livestock case in this epidemic on Mayotte. Eq 3A represented the probability of commune *i* avoiding the infection until *t*_*i*_−1 and becoming infected on *t*_*i*_. Eq 3B was the probability of commune *j* avoiding the infection during the epidemic. Given that the first four livestock cases were recorded in three communes within ten days in early December 2018, several weeks before the epidemic took off in January 2019, RVFV was assumed to have been introduced in these three communes, referred to as seeding communes.

We estimated parameters (*ψ*, *σ*, *q*, *ω* and *s*) within a Bayesian Markov Chain Monte Carlo (MCMC) framework. We used a single-component random-walk Metropolis-Hastings (SCMH) algorithm [30] to express the above likelihood function explicitly. The models were run by custom-built codes in R.3.4.2 [31], and those with the same time-lag and time-dependency assumptions were compared with one another based on deviance information criterion (DIC) values. Additionally, for the best model (see Model evaluation below), we compared for each commune the rate of infection resulting from a given transmission route (e.g. livestock movements) with the background rate of infection. For this, we randomly sampled parameter values from their joint posterior distributions and computed the rate of infection for each commune on the day their first livestock case was confirmed. We then computed the proportions of simulations for which the rate of infection resulting from a given transmission route was higher than the background rate of infection. In each simulation (i.e. for each set of parameters sampled from the joint posterior distribution), we also randomly assigned a source of infection to each commune by simulating a binomial trial, with a probability of success equal to *p*_*i*,*d*_/*λ*_*i*,*d*_. Finally, we performed a posterior predictive check by comparing the observed and simulated temporal sequence of communes’ infection. In each simulation, we introduced RVFV into the three seeding communes and simulated disease transmission between communes based on parameter values randomly sampled from their joint posterior distributions.

#### Association between human and livestock Rift Valley fever epidemic patterns

We investigated the spatio-temporal association between livestock and human cases, to test the hypothesis that the risk of infection for humans was higher if the virus was present in nearby livestock populations. The epidemic was divided into nine 4-week epidemic periods. With this 4-week interval, among commune-period combinations that experienced at least one human case, the number of human cases per commune and period was low, with a median of one case (interquartile range (IQR): 1–2.3) and a maximum value of 11 cases. The infection pressure on humans residing in a commune from a livestock-infected commune was formulated in 12 different ways. First, we assumed that it (a) decreased exponentially as the distance between the two communes increased (‘distance model for human infection’, Model H.1), (b) depended on livestock density in a livestock-infected commune (‘livestock density model for human infection’, Model H.2) or (c) was not influenced by those factors (‘a null model’, Model H.3), as a baseline comparison. Each of these 3 models was fitted with or without accounting for a possible delay between the onset of symptoms and sampling of livestock cases (3 × 2 = 6 models), and each of these 6 models was further evaluated assuming time-dependent or time-independent rates of infection (6 × 2 = 12 models). The model formulations are presented below.

For all models, the observed number of humans infected by RVFV in commune *i* during the period *t*, *τ*_*i*,*t*_, was simulated through a Poisson process:

$$\tau_{i,t}∼P\left(\delta_{i,t}\right)$$ (4)

$$\delta_{i,t}=\pi_{i}\varepsilon_{t}\eta_{i,t}$$ (5)

*δ*_*i*,*t*_ was the expected number of human cases in commune *i* during period *t* and formulated as a product of *π*_*i*_ (i.e. the size of the human population in commune *i*), *ε*_*t*_ (i.e. the daily baseline rate of human infection during period *t* on the island), and *η*_*i*,*t*_ (i.e. a measure of the relative rate of human infection in commune *i* in period *t*). *η*_*i*,*t*_ was expressed in 3 different ways (Eqs 6A, 6B or 6C).

When *η*_*i*,*t*_ depended on the spatial proximity from livestock-infected communes (Model H.1), the rate of humans infection in commune *i* from livestock-infected commune *j* decreased exponentially at a rate *α* as the Euclidean distance D_*i*,*j*_ between the geometric centroids of both communes increased. Therefore, with *θ*_*j*,*t*_ defined as the number of days during which commune *j*’s livestock population was infected and infectious in period *t*, *η*_*i*,*t*_ was expressed as:

$$\eta_{i,t}=\sum_{j=1}^{j=17}(e^{-\alpha D_{i,j}}\theta_{j,t})$$ (6A)

Secondly, when *η*_*i*,*t*_ was assumed to depend on the livestock density in livestock-infected communes (Model H.2), the rate of human infection in commune *i* from livestock-infected commune *j* was assumed to increase with livestock density in commune *j* (*γ*_*j*_) to the power of *β*. *η*_*i*,*t*_ was expressed as:

$$\eta_{i,t}=\sum_{j=1}^{j=17}\left(\gamma_{j}^{\beta }\theta_{j,t}\right)$$ (6B)

Finally, in the null model (Model H.3),

$$\eta_{i,t}=1.$$ (6C)

In Models H.1–3, the daily baseline rate was fixed during the entire epidemic (*ε*_*t*_ = *ε*, ‘time-independent models’) or allowed to vary with the considered time period *t* (‘time-dependent models’). The three parameters (*ε*_*t*_, α, and β) were estimated by using an MCMC simulation in JAGS [32] in R.3.4.2 [31]. JAGS was used, instead of the SCMH algorithm, because *τ*_*i*,*t*_ could be modelled as a function of *δ*_*i*,*t*_ directly in JAGS. In order to assess the impact of a delay between the onset of symptoms and sample collection in livestock cases on the model results, we fitted the models with all sampling dates moved backwards by 2 weeks, the estimated maximum delay (‘time-lag models’). The parameters and variables in the above models are summarized in Table 2. We compared the models with the same time-lag and time-dependency assumptions with one another, based on DIC values, and performed a posterior predictive check on the best model to assess its goodness of fit. For each commune *i* and period *t*, we randomly sampled model parameters from the joint posterior distribution, and we simulated the number of human cases based on a Poisson process. We repeated this procedure 10,000 times and compared the observed and simulated number of human cases.

**Table 2:** Parameters and variables in the models investigating factors associated with the epidemic in humans.

Model	Parameter or variable		
	Notation	Description	Prior
Models H.1–3	*τ*_*i*,*t*_	Observed number of humans infected by RVFV in commune *i* during the period *t*	NA
	*δ*_*i*,*t*_	Expected number of human cases in commune *i* during period *t*	NA
	*π*_*i*_	Human population size in commune *i*	NA
	*ε*_*t*_	Daily baseline rate of human infection in period *t* on the island	Normal (0, 10,000)
	*η*_*i*,*t*_	Relative rate of human infection in commune *i* in period *t*	NA
Models H.1–2	*θ*_*i*,*t*_	Number of days commune *i*’s livestock population was infected and infectious in period *t*	NA
Distance (Model H.1)	D_*i*,*j*_	Euclidean distance between communes *i* and *j*	NA
	*α*	Exponent for spatial proximity	Normal (0, 10,000), I(0,) ^a^
Livestock density (Model H.2)	*γ*_*i*_	Livestock density in commune *i*	NA
	*β*	Exponent for livestock density	Normal (0, 10,000), I(0,) ^a^

a Priors for *α* and *β* were left-truncated.

NA = not applicable

#### Model evaluation

All models were run with two chains and iterated up to the point where convergence was considered achieved for all unknown parameters based on a visual inspection of trace plots and the Gelman–Rubin convergence diagnostic (<1.01) [33]. Weakly informed priors were used for all unknown parameters (Tables 1 and 2), and the first 10,000 iterations were discarded from the posterior distributions. A posterior predictive check was performed to assess the goodness of fit. For each objective, when comparing any two models, we considered that a model explained the data substantially better than another model if their DIC difference was greater than five [34]. We selected the model whose DIC difference from its null model was greatest as the best model.

## Results

### Livestock and human epidemic patterns

Livestock cases (n = 165) were identified through the report of abortions (n = 116, 70.3%) and other clinical signs suggestive of RVFV (e.g. pyrexia) (n = 49, 29.6%) from 109 farms. The number of livestock cases per farm showed a highly right-skewed distribution, with only one livestock case detected in 76.1% of affected farms (n = 83) (Fig C in S1 Text). About half of the human cases (n = 70, 49.0%) reported living or working close to animals, and 60.1% (n = 86) reported recent direct contacts with animals or animal fluids.

**Fig 2:**
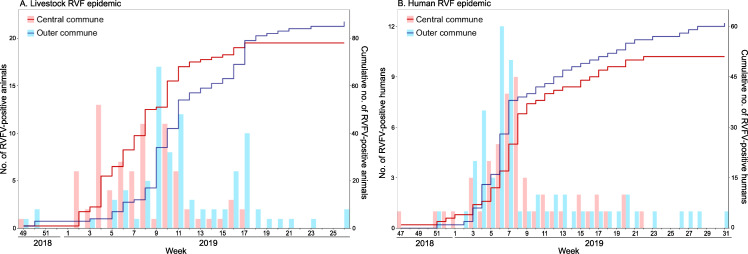
**Weekly number of Rift Valley fever virus (RVFV) RT-PCR positive (A) animals and (B) humans in central or outer communes.** The bar plots show the weekly number of RVFV-positive cases (left y-axis), and the solid lines their cumulative distributions (right y-axis). Central communes are colored in red and outer communes in blue.

**Fig 3:**
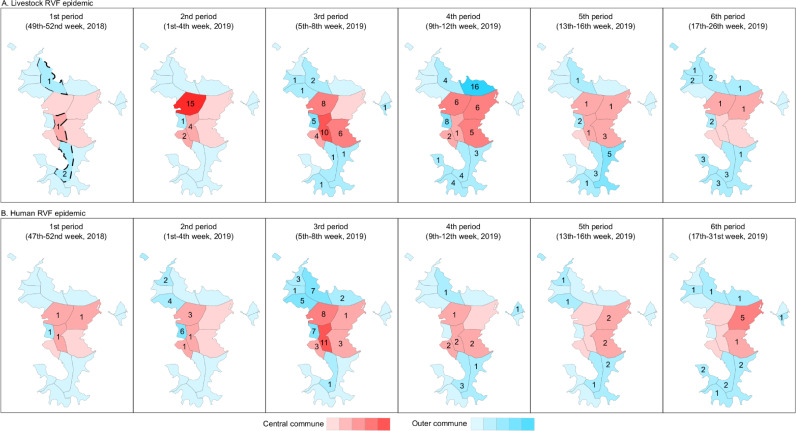
**Spatio-temporal pattern of Rift Valley fever virus (RVFV) RT-PCR positive (A) animals and (B) humans.** The epidemic was divided into five 4-week periods, and a sixth period comprised the last 15 weeks of the epidemic. The numbers on the map show the number of RVFV RT-PCR positive cases reported in each commune. Central communes are colored in red and outer communes in blue, with a darker shade representing a higher number of RVFV RT-PCR positive cases. Communes where RVFV was assumed to have been introduced into livestock (‘seeding communes’) are represented with a dashed border in the first map of Fig 3A.

Communes were classified as *central* (n = 5) and *outer* (n = 12) based on the structure of the 2007–14 livestock movement networks, with those movements being much more frequent among central, and from central to outer communes, than from outer communes to other outer and central communes [11]. During the 2018–19 epidemic, the number of livestock cases was slightly higher in outer (n = 87, 52.7%) than central communes (n = 78, 47.3%). However, most cases in central communes were reported earlier than those in outer communes. During the first half of the epidemic (i.e. the first 50% of livestock cases), central communes accounted for 61.0% of cases, whereas in the second half of the epidemic, outer communes accounted for 66.3% of cases (Figs 2A and 3A). A similar pattern was observed at farm-level, with farm cases being defined as farms with at least one livestock case (Figs D and E in S1 Text). Information on the commune of residence was available for 112 out of 143 human cases (73.8%). The overall number of human cases was also slightly higher in outer (n = 61, 54.5%) than central communes (n = 51, 45.5%). However, infections in central communes did not seem to precede infections in outer communes (Figs 2B and 3B).

### Role of the Mayotte livestock movement network in the livestock epidemic

Convergence was achieved for all models (Fig F in S1 Text). Table 3 shows the DIC values of different models. The weighted livestock movement models had the lowest DIC value under all model formulations (i.e. time-dependent and time-independent infection rates, with or without a time lag). In particular, the model with time-independent infection rates and a lag between viral incursion and transmission in a commune had the greatest DIC difference from its null model and was therefore chosen as the best model. Additionally, the spatial proximity model with time-independent infection rates and a time-lag in transmission explained the livestock case data considerably better than its null model, although it was not the case for the other formulations of the spatial proximity models. Finally, for all models, the difference in DIC from the null model was reduced when infection rates could vary over time (‘time-dependent models’).

**Fig 4:**
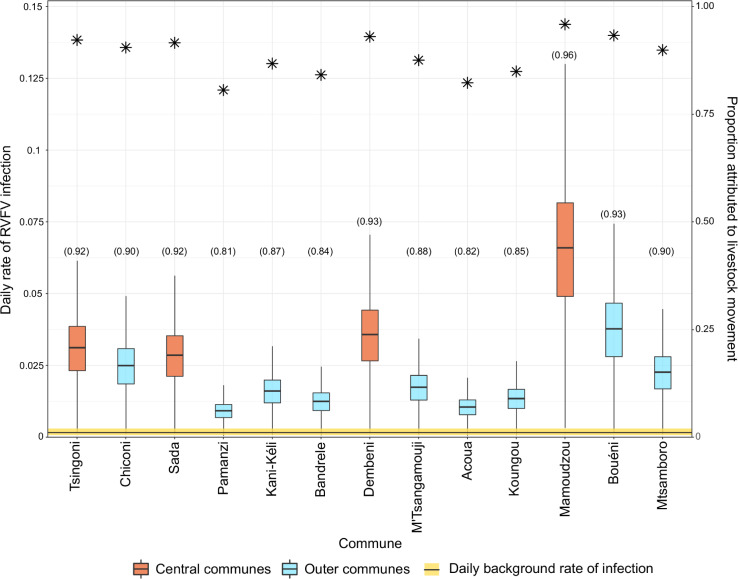
Daily rate of infection per commune estimated from the best livestock movement model. It shows the results of the weighted, time-independent and time-lag livestock movement model. Boxplots show the posterior distributions of the daily rate of infection from livestock movements (red and blue boxplots) and the background daily rate of infection (yellow horizontal bar) on the observed infection day of each commune. Asterisks represent the proportion of simulations where RVFV introduction into a commune was due to livestock movements rather than other routes (i.e. background risk) (y-axis). Numbers in brackets along x-axis labels correspond to the proportion of iterations in which the daily rate of RVFV infection exerted by livestock movements on each commune on the day they reported their first livestock case was greater than the background daily rate of infection.

On the day that communes had their first livestock case, the estimated force of infection resulting from livestock movements was greater than the background force of infection, especially in central communes (Fig 4). In a posterior predictive check, the model reproduced reasonably well the temporal sequence of infection among communes (Fig G in S1 Text).

**Table 3:** Models assessing factors associated with the livestock epidemic.

Model	Time-independent infection rates		Time-dependent infection rates	
	DIC	No. of parameters	DIC	No. of parameters
Model L.1.1 –Weighted livestock movement	135.0 ^b^ (131.0^a^^,^ ^b^)	2	134.1 (134.9)	6
Model L.1.2 –Unweighted livestock movement	138.2 (137.2^b^)	2	138.3 (139.0)	6
Model L.2 –Spatial proximity	138.5 (133.9^b^)	2	135.3 (135.5)	6
Model L.3 –Livestock density	139.5 (140.3)	2	137.2 (137.5)	6
Model L.4 –Null model	143.1 (143.1)	1	137.8 (137.5)	5

DIC = deviance information criterion

DIC values in brackets are from the time-lag models (i.e. delay between viral incursion and transmission in a commune)

a The best model with the greatest DIC difference from its null model (see Table A in S1 Text for the parameter estimates of the best model)

b The DIC difference from the null model is >5

### Association between human and livestock epidemic patterns

Convergence was obtained for all models. Models assuming that the rate of human infection depended on the livestock density in livestock-infected communes (Model H.2) did not explain the spatio-temporal pattern of human RVF cases better than the null model. In contrast, the models assuming that the rate of human infection was a function of the distance to livestock-infected communes (Model H.1) had substantially lower DIC values than other models (DIC difference >20 for all model formulations). These patterns were not affected by considering time-independent or time-dependent rates of human infection, and by accounting or not for a plausible delay in the detection of livestock cases (Table 4). The distance model accounting for time-dependent infection rates and without delay in the detection of livestock cases had the greatest DIC difference from its null model and was therefore chosen as the best model (Table 4).

**Table 4:** Models assessing the association between the livestock and human epidemics.

Model	Time-independent infection rates		Time-dependent infection rates	
	DIC	No. of parameters	DIC	No. of parameters
Model H.1 –Distance	463.7^b^ (446.9^b^)	2	323.9^a^^,^ ^b^ (334.4^b^)	10
Model H.2 –Livestock density	511.9 (485.6)	2	386.0 (386.0)	10
Model H.3 –Null model	484.4 (484.4)	1	386.1 (386.1)	9

DIC = deviance information criterion

DIC values in brackets are from the time-lag models (i.e. delay in the detection of livestock cases)

a The best model with the largest DIC difference from its null model (see Table B in S1 Text for the parameter estimates of the best model)

b The DIC difference from the null model is >5

**Fig 5:**
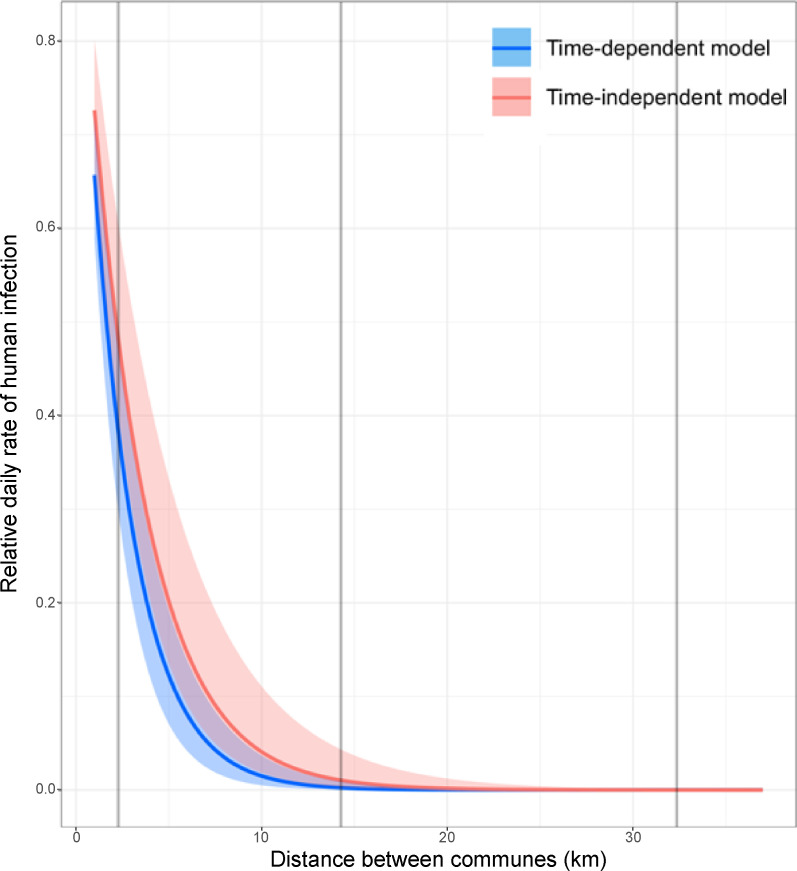
Relative daily rate of human infection in a commune as a function of the distance from a livestock-infected commune. Mode (solid line) and 95% highest density intervals (shaded areas) are shown. The vertical lines represent the minimum, median and maximum Euclidean distances between the centroids of Mayotte communes, respectively (from left).

While the time-dependent formulation of the distance model (Model H.1) predicted well the observed number of RVF-positive human cases in each epidemic period, the posterior predictive check of the time-independent formulation of this model showed discrepancies between observed and predicted values (Fig H in S1 Text). However, as the distance from livestock-infected communes increased, the infection rate decreased similarly in both time-dependent and time-independent models, at a rate of α = 0.43 (median, 95% highest density interval (HDI): 0.33–0.52) and 0.31 (median, 95% HDI: 0.23–0.40), respectively (Fig 5). At such rates, compared to the daily rate of human infection exerted by infected livestock in the same commune, the daily rate of human infection exerted by infected livestock from another commune decreased by 61.7% with the time-dependent, and by 51.9% with the time-independent models, at a distance of 2.3km, the shortest distance between any two commune centroids, and became negligible at a distance of 14.3km, the median distance between any two communes (Fig 5).

## Discussion

Our study shows that during the 2018–2019 RVF epidemic in Mayotte, the temporal sequence of RVFV introduction into communes’ livestock populations was associated with the historic patterns of livestock movements and, potentially less strongly, with spatial proximity from communes with infected livestock. Also, the spatio-temporal pattern of RVFV infection in humans was associated with spatial proximity from communes with infected animals. Those findings suggest that the analysis of livestock movement networks could be used to identify epidemiological units at high risk of becoming infected and spreading RVFV in both animals and humans.

Previous transmission models based on livestock movement data have suggested a contribution of livestock movements to the maintenance of RVFV circulation in endemic settings [16,17]. Livestock movements, through the import of infectious animals, were already shown to be a likely route of RVFV introduction into Mayotte [7]. Our findings support the hypothesis that livestock movements may also drive the epidemic spread of RVFV within a naïve host population. Approximately 95% of livestock was estimated to be susceptible to RVFV infection before the 2018–19 epidemic [35], providing suitable conditions for a large RVFV epidemic. Therefore, the introduction of infected animals into a commune’s susceptible livestock population could lead to the amplification of RVFV in both livestock and mosquitoes, which could then pose an infection risk to humans in the area, as shown by the association between the livestock and human RVF epidemics. However, it should also be noted that livestock movements were unlikely the only factor shaping the transmission pattern of RVFV on the island. When the spatial proximity model accounted for a time lag, its DIC value also became substantially lower than the null model and marginally higher than the livestock movement model. Considering the time required for the virus to amplify in a mosquito population after its introduction and possible dispersal of infected mosquitos to neighboring communes, this suggests that the observed pattern could have also been influenced by mosquitoes’ dispersion. Indeed, the influence of westerly wind gradually became stronger at the early stage of the epidemic, coinciding with the eastward expansion of RVFV [36]. NDVI was relatively similar across the island, and entomological surveys conducted in Mayotte were consistent with our assumption that spatial variations in RVFV vectors may be limited [37,38]. However, entomological data on potential RVFV vectors still remain limited, meaning that the RVFV vectors’ dynamics is yet to be elucidated in Mayotte [37,38]. Therefore, we cannot exclude the possibility that other ecological factors, which are heterogeneously distributed across the island impact on mosquito population dynamics, result in spatial variations in mosquito abundance and eventually influence RVFV transmission dynamics.

Contrary to livestock, humans were not affected in central communes earlier than in outer communes. Nonetheless, the model suggested that RVFV infection pressure on humans was associated with the distance from livestock-infected communes, with the infection pressure rapidly decreasing with the distance. This may reflect mobility patterns on the island. Indeed, communes reported for human cases were the commune of residence, not the commune of workplace. Yet, early in the epidemic, livestock cases were clustered in central communes where many people commute to, from outer communes. Contacts with infected animal tissues or fluids, or bites by mosquitoes fed on infected animals may have thus occurred at the workplace, rather than the commune of residence. Interestingly, the spatial distribution of RVF human cases contrasted drastically with the spatial distribution of COVID-19 cases, which were clustered in Mamoudzou, the capital of Mayotte [39], highlighting different primary transmission routes for those two diseases.

The livestock- and human-infection models associated with the lowest DIC value were time-independent and time-dependent, respectively. This is likely due to the different model outcomes: the date of viral incursion in a commune’s livestock population, and the number of human cases in a commune. While the dates at which livestock were first detected as infected in all but one commune spread over several months, around half of the human cases were confirmed only over a month. Therefore, allowing the infection rate to vary over time was more likely to impact substantially the goodness of fit of the human than the livestock-infection model.

Our results suggest that understanding the structure of livestock movement networks could help identify high-risk epidemiological units and therefore benefit RVF surveillance, from both animal and public health perspectives. As already emphasized in an earlier study [11], animal health surveillance should initially be targeted to central communes. In the event of an RVF incursion on the island, communication messages could particularly target people living or working in the affected and high-risk areas, encouraging them to practice good hygiene when handling animal fluids and tissues, and to seek medical advice when they have dengue-like symptoms [10]. Metras et al. [18] suggested that targeting hypothetical vaccination efforts on livestock, rather than on humans, would be most effective to reduce the burden of RVF human infections. Our results could support the design of such vaccination programs by identifying livestock populations where vaccination should be prioritized.

This study had several limitations. First, considerable under-reporting was possible for livestock and human cases. For livestock, subclinical infections, which were likely to account for most infections, as well as infections associated with mild clinical signs, would have been missed by farmers. Moreover, while people were tested by RT-PCR on the day of their medical consultation, animals have been sampled several days after clinical signs suggestive of an RVFV infection were reported. Therefore, it was also likely that most infected animals were no longer viremic at the time of testing and therefore missed. Under-reporting of livestock cases was also likely to depend on farms’ accessibility and whether they had a declared state veterinarian. In Mayotte, while some farms were not easily accessible by road, they were scattered across the island and not clustered in particular communes. Also, as mentioned above, central and outer communes had similar proportions of farms with a declared state veterinarian. These suggest that the level of under-reporting, although not negligible, may have been similar across the island, and the observed temporal sequence of communes’ infection may still reflect the actual epidemic patterns. For humans, under-reporting was also possible as RVFV infections may be asymptomatic or associated with a mild illness, as observed in a previous epidemic [40]. However, for the past ten years, people with dengue-like symptoms have been tested for RVFV when visiting local healthcare facilities across the island [10], suggesting that the observed human epidemic curve may have also captured the actual spatio-temporal pattern of human cases. For both livestock and human cases, if the level of biases caused by the above factors was more severe in some communes, the observed associations could have been indeed the result of differential bias.

Another limitation was that dates of infection of livestock and humans were not reported. Since people were tested on the day of medical consultation, the recorded dates were likely to reflect the timing of symptom onsets. For animals, sampling dates were available, but these may not correspond to the onset of clinical signs, due to the time possibly taken by farmers to report a suspected infection, and by state veterinarians to visit farms. However, this is unlikely to change the interpretation of our findings because the model accounting for such delays still explained the spatio-temporal pattern of human cases better than the other models tested.

Third, we modelled livestock movements during the 2018–19 epidemic based on data for 2007–14. However, given that the overall structure of the livestock movement network was stable between 2007 and 2014 [11] and the relative importance of communes in terms of their numbers of livestock did not change between 2010 and 2015, the average network structure over the 2007–14 period was therefore likely to be a good approximation for the network structure in 2018–19. Also, there has not been any particular event known to the authors which might have altered livestock movement patterns in 2018–19.

Fourth, we assumed that a commune remained infected throughout the epidemic once the first livestock case was reported, and the level of infectiousness of an infected commune did not vary over the study period, or if it did, it was similar, at a given time point, in all infected communes. Yet, viral circulation likely varied over time within a given commune, and also between communes, as a result, for instance, of variations in intra-commune movement patterns. To account for temporal variations in the rate of infection, we also considered time-dependent rates of infection. However, those models did not change the interpretation of the results, nor did they account for within-commune transmission dynamics. Thus, the availability of prevalence data in both livestock and humans could allow the modelling of RVF dynamics at a higher resolution. Finally, no entomological data were available to assess the potential role of mosquitoes in the 2018–19 RVF epidemic, and variations in RVFV vector abundance across the island. While livestock movements were shown to be associated with RVFV spread across Mayotte, vector transmission was also likely to have played a role in shaping the dissemination of the virus across the island, as discussed above in relation to the spatial proximity model. The availability of entomological data would allow us to compare the relative importance of vectors and animal movements in the spatial dissemination of the virus.

In conclusion, our study shows that the livestock movement network and, potentially to a lesser extent, spatial proximity from communes with infected livestock influenced the spatio-temporal pattern of the 2018–19 RVF epidemic in livestock. Our study also shows the spatio-temporal association between human and livestock RVF epidemics, highlighting the need for One Health approaches in coordinating surveillance programs in both livestock and human populations.

## Supporting information

S1 Text Supplementary tables and figures. (DOCX)
