# Supplementary material for: The role of livestock movements in the spread of Rift Valley fever virus in animals and humans in Mayotte, 2018–19
Source: PLoS Negl Trop Dis. 2021 Mar 8;15(3):e0009202. doi: 10.1371/journal.pntd.0009202 (PMC7939299; doi:10.1371/journal.pntd.0009202)
Supplement: S1 Text — (DOCX) [file pntd.0009202.s001.docx]

**The role of livestock movements in the spread of Rift Valley fever virus in animal and human populations in Mayotte, 2018-19**

Younjung Kim, Raphaëlle Métras, Laure Dommergues, Chouanibou Youssouffi, Soihibou Combo, Gilles Le Godais, Dirk U. Pfeiffer, Catherine Cêtre-Sossah, Eric Cardinale, Laurent Filleul, Hassani Youssouf, Marion Subiros, and Guillaume Fournié

**Supplementary tables and figures**

**Table A.** Parameter estimates from weighted time-independent livestock movement model for the epidemic in livestock

| Model | Parameter | Median (95% HDI) | |
| --- | --- | --- | --- |
|  |  | without a time-lag | with a time-lag |
| Model L.1.1 –  weighted time-independent livestock movement | $\psi$ | 0.44 (0.14 – 0.76) | 0.80 (0.34 – 1.36) |
|  | $q$ | 0.21 (0 – 0.84) | 0.17 (0 – 0.67) ×10^-2^ |

**Table B.** Parameter estimates from time-dependent distance model for the epidemic in humans

| Model | Parameter | Median (95% HDI) | |
| --- | --- | --- | --- |
|  |  | without a time-lag | with a time-lag |
| Model H.1 –  time-dependent distance | $\varepsilon_{1}$ | 3.82 (0.12 – 11.06) | 1.61 (0.04 – 4.73) |
|  | $\varepsilon_{2}$ | 4.24 (1.30 – 8.35) | 3.18 (0.94 – 6.23) |
|  | $\varepsilon_{3}$ | 1.36 (8.78 – 19.06) | 10.53 (6.80 – 14.69) |
|  | $\varepsilon_{4}$ | 7.00 (4.55 – 9.82) | 5.52 (3.62 – 7.70) |
|  | $\varepsilon_{5}$ | 1.41 (0.62 – 2.36) | 1.46 (0.66 – 2.45) |
|  | $\varepsilon_{6}$ | 1.40 (0.62 – 2.33) | 1.41 (0.63 – 2.37) |
|  | $\varepsilon_{7}$ | 0.98 (0.37 – 1.77) | 1.01 (0.38 – 1.83) |
|  | $\varepsilon_{8}$ | 0.34 (0.04 – 0.83) | 0.35 (0.04 – 0.85) |
|  | $\varepsilon_{9}$ | 0.47 (0.09 – 1.03) | 0.48 (0.09 – 1.05) |
|  | $\alpha$ | 0.43 (0.33 – 0.52) | 0.45 (0.34 – 0.56) |

**
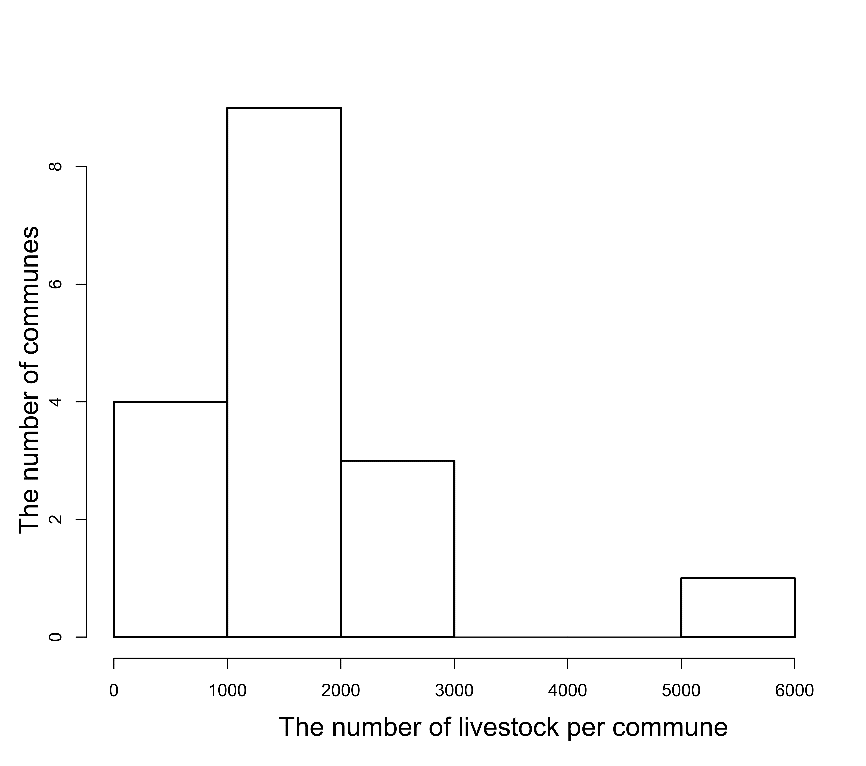
**

**Fig A.** Number of livestock (i.e. cattle, sheep, and goats) per commune from the 2010 agricultural census.

**
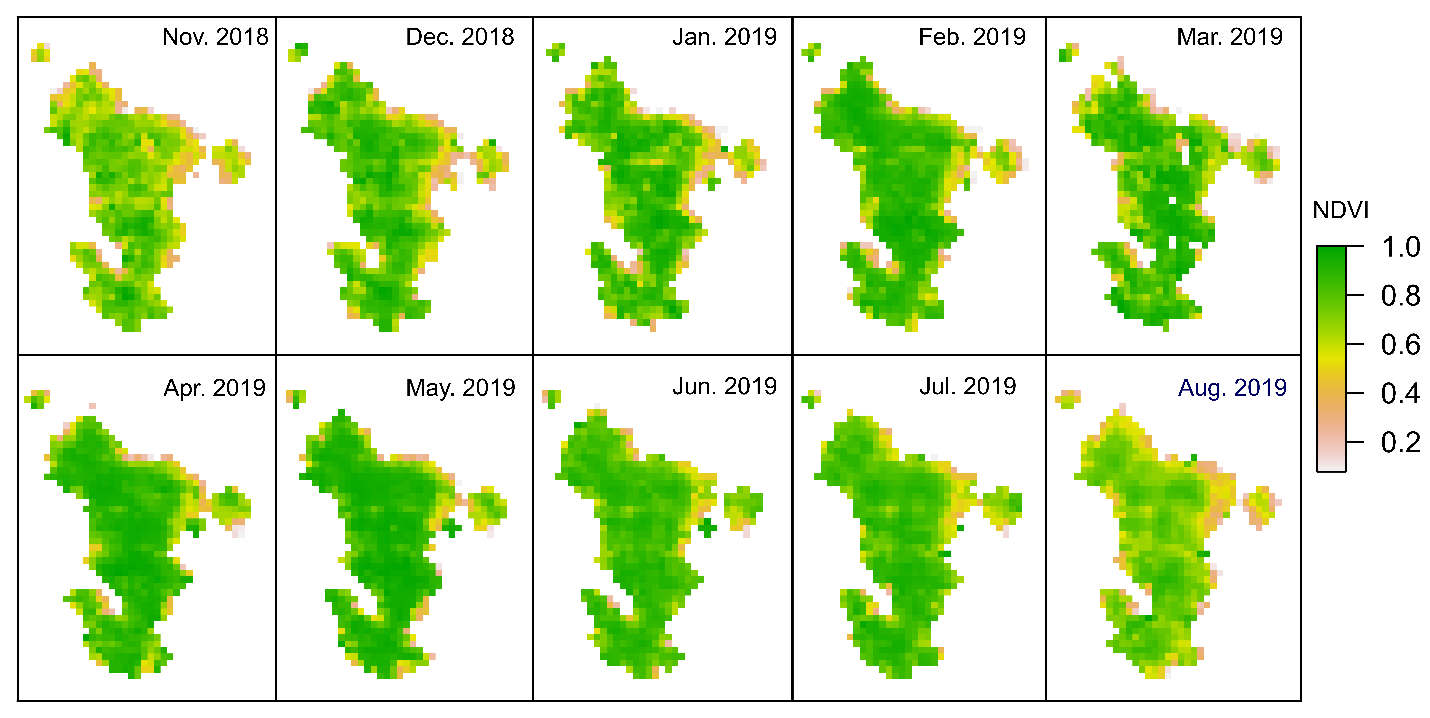
**

**Fig B.** Distribution of vegetation and water presence based on Normalized Difference Vegetation Index (NDVI) values during the study period. NDVI values were sourced from MODIS/Terra Vegetation Indices Monthly L3 Global 1 km SIN Grid (MOD13A3).

**
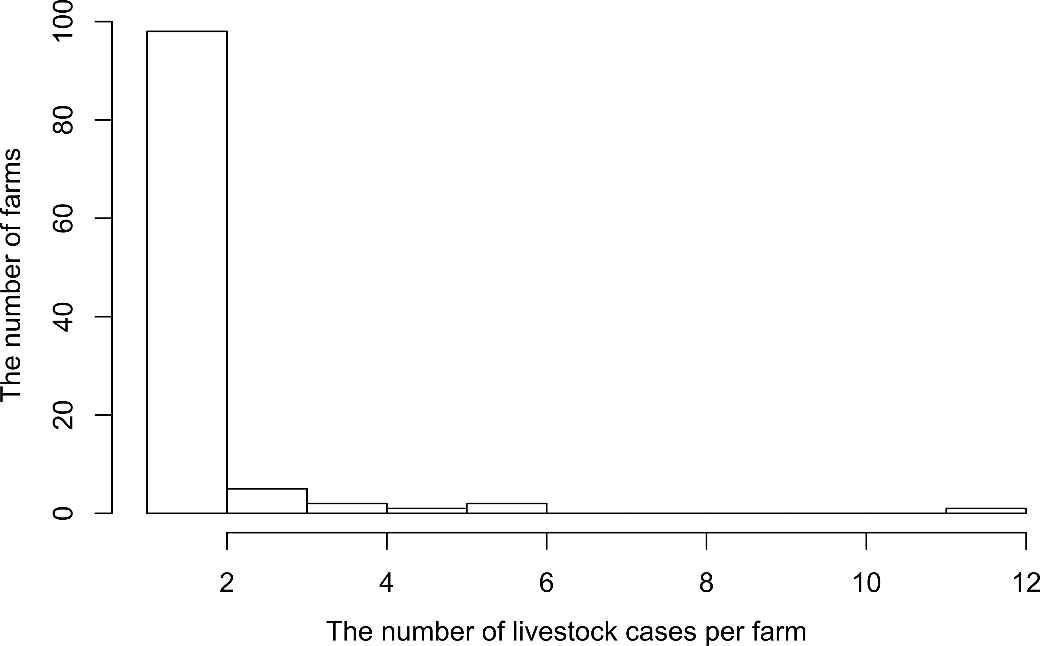
**

**Fig C.** Distribution of the number of livestock cases per farm in the 2018–19 Rift Valley fever (RVF) epidemic in Mayotte. A total of 165 livestock cases were identified from 109 farms.


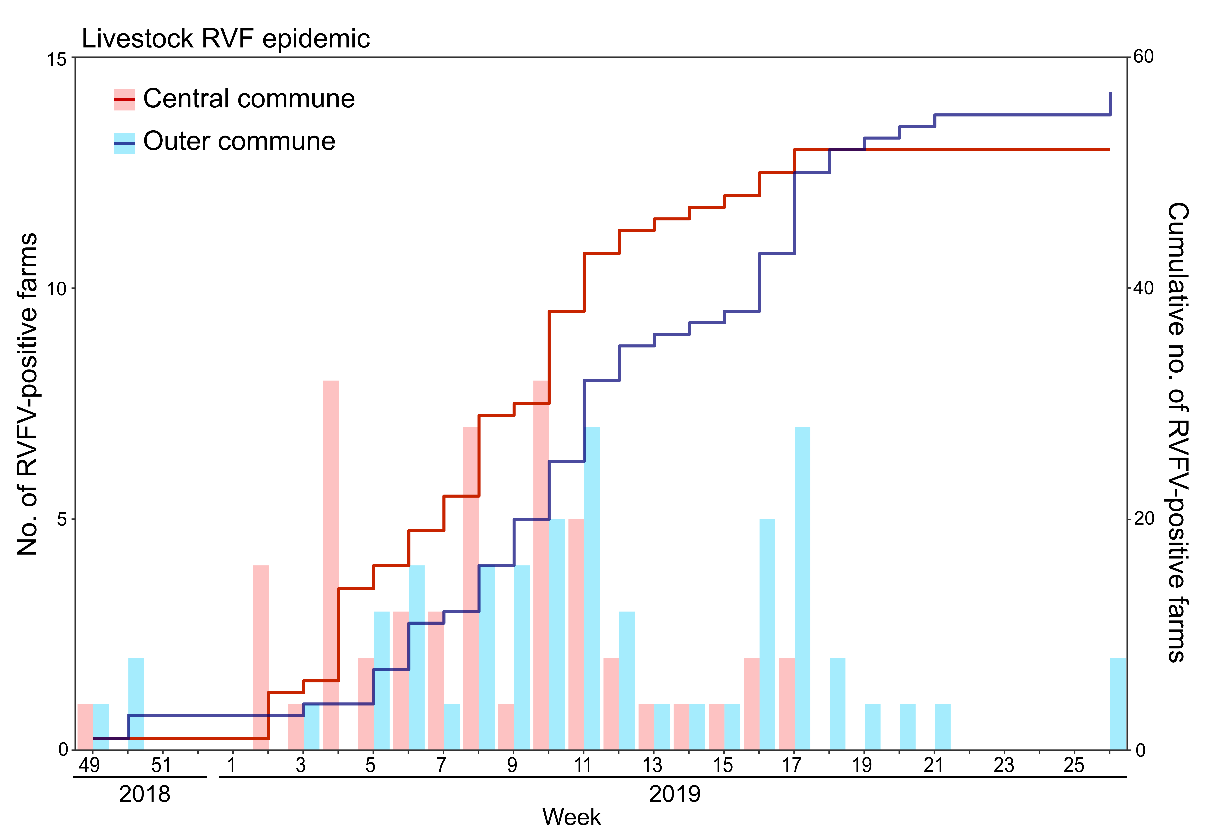


**Fig D.** Weekly number of Rift Valley fever virus (RVFV) RT-PCR positive livestock farms per commune cluster. The bar plot shows the number of RVFV-positive livestock farms (left y-axis), and the solid lines show their cumulative distributions (right y-axis). Central communes are colored in red and outer communes in blue.


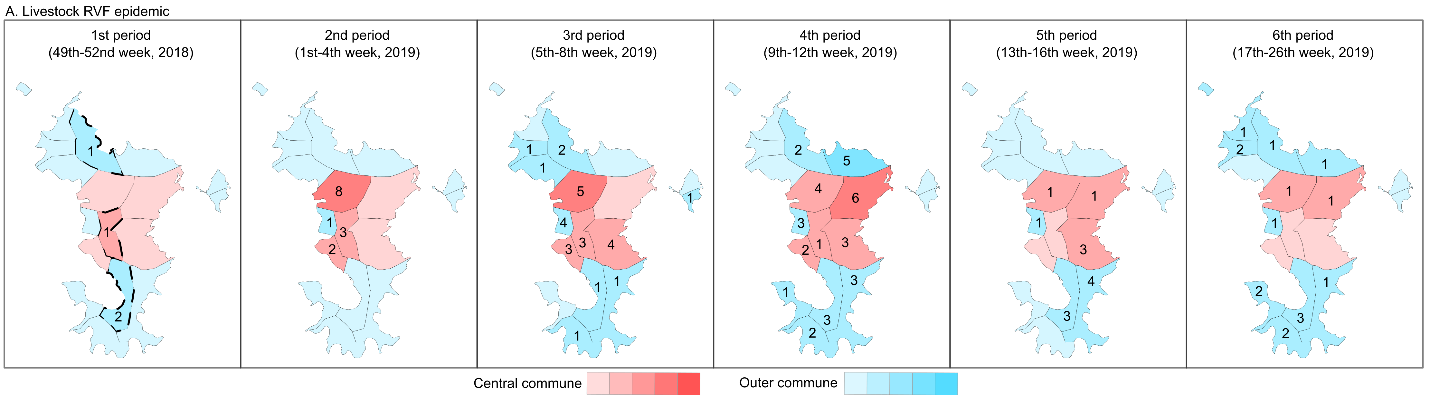


**Fig E.** Spatio-temporal pattern of Rift Valley fever virus (RVFV) RT-PCR positive livestock farm cases. The epidemic was divided into six different phases. The numbers on the map show the number of RVFV RT-PCR positive livestock farm cases reported in each commune. Central communes are expressed by red, and outer communes in blue, with a darker shade representing a higher number of RVFV RT-PCR positive livestock farm cases.


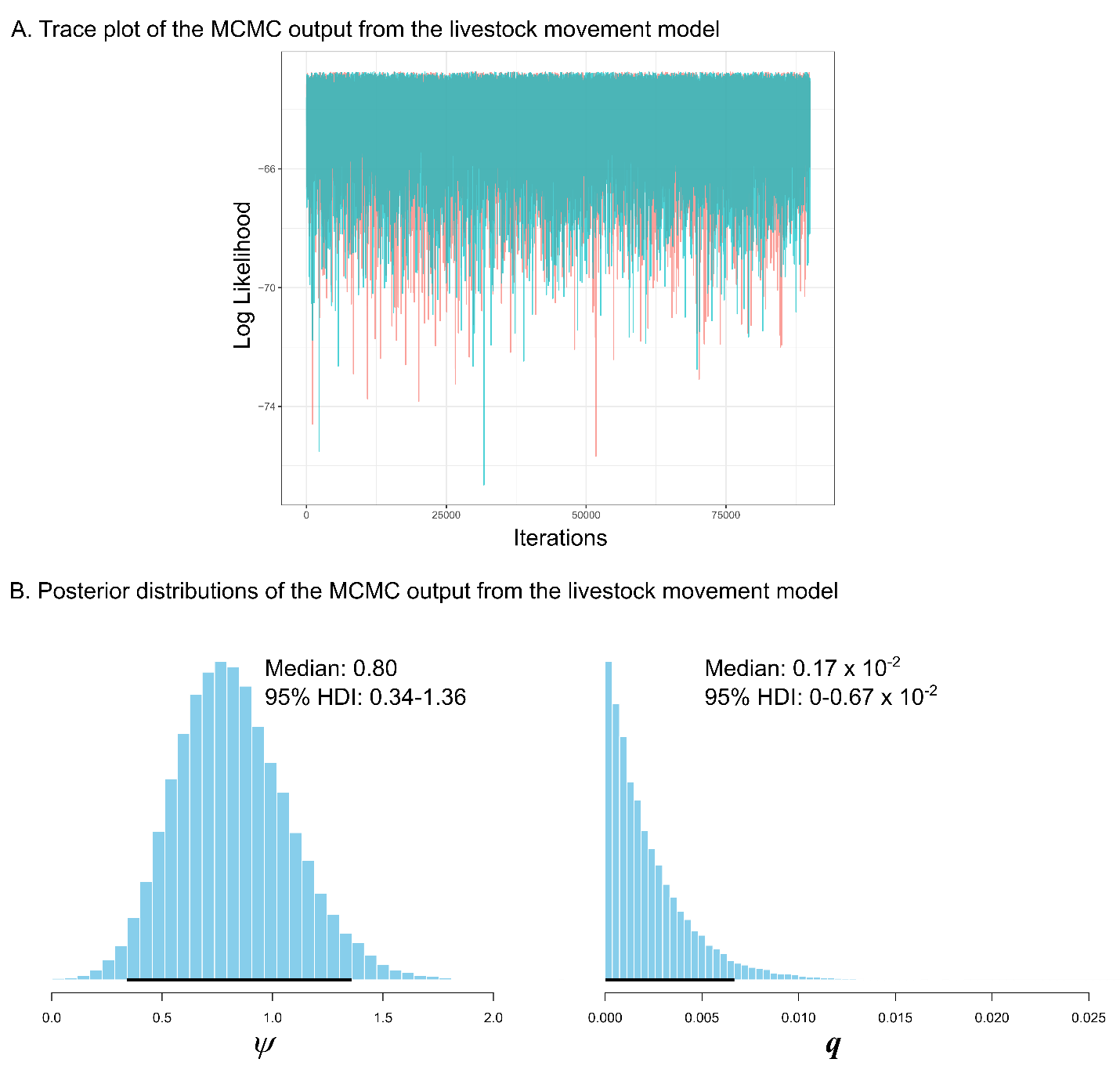


**Fig F.** Markov Chain Monte Carlo (MCMC) output from the weighted, time-independent, and time-lag livestock movement model. **(A)** Trace plot of the log-likelihood for the temporal sequence of infection of Mayotte communes’ livestock populations. Different colors represent chains with different starting values. **(B)** Posterior distribution of $\psi$, probability of an animal moved from a livestock-infected commune being infected and transmitting the virus to the livestock population of a commune into which it was introduced, and $q$, background force of infection. Thick black horizontal lines represent 95% highest-density intervals (95% HDI). The first 10,000 iterations were discarded.


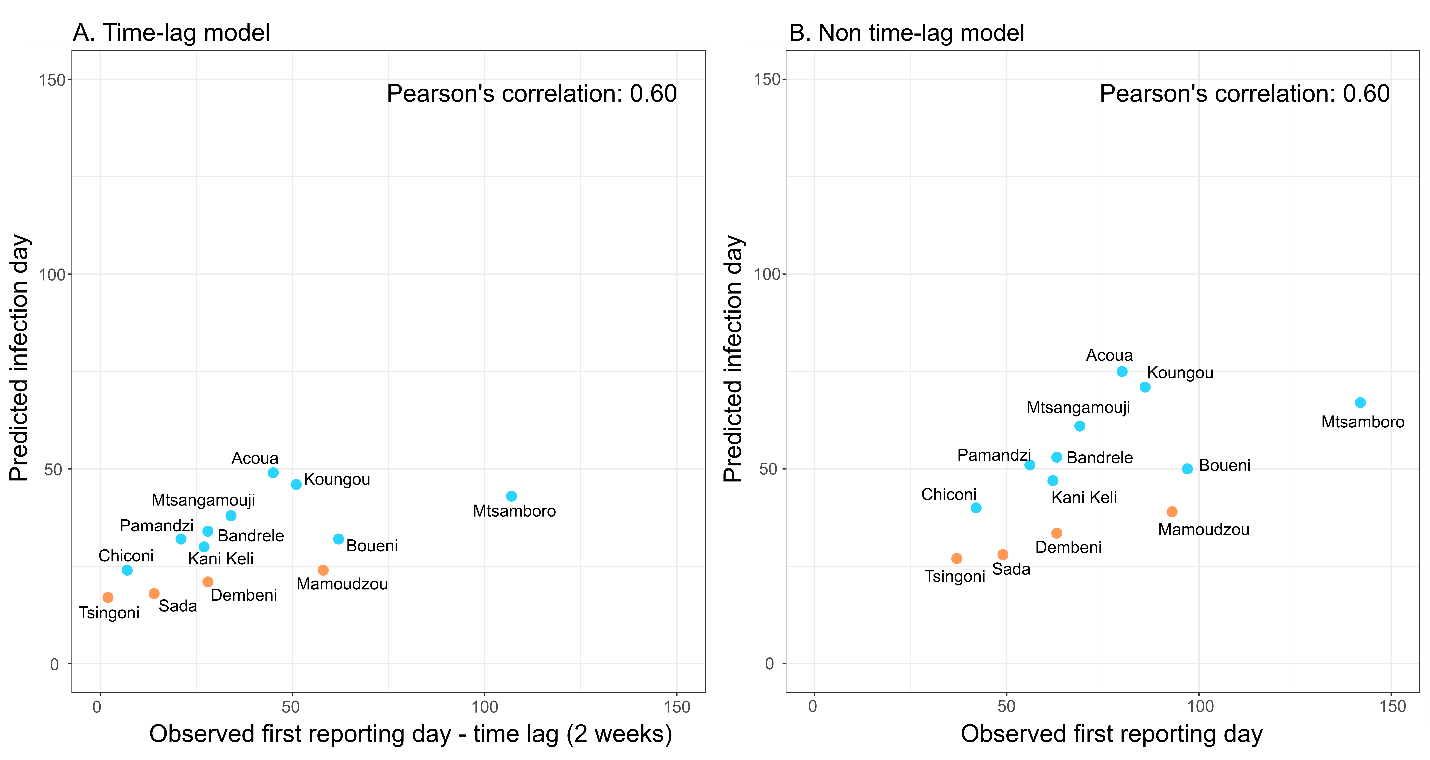


**Fig G.** Posterior predictive check of the weighted and time-independent livestock movement models with **(A)** and without **(B)** a time lag in commune infection and infectiousness. Each point corresponds to a commune (orange: central communes, blue: outer communes). The predicted infection day for a commune (y-axis) was the median of simulated infection days for this commune.

**
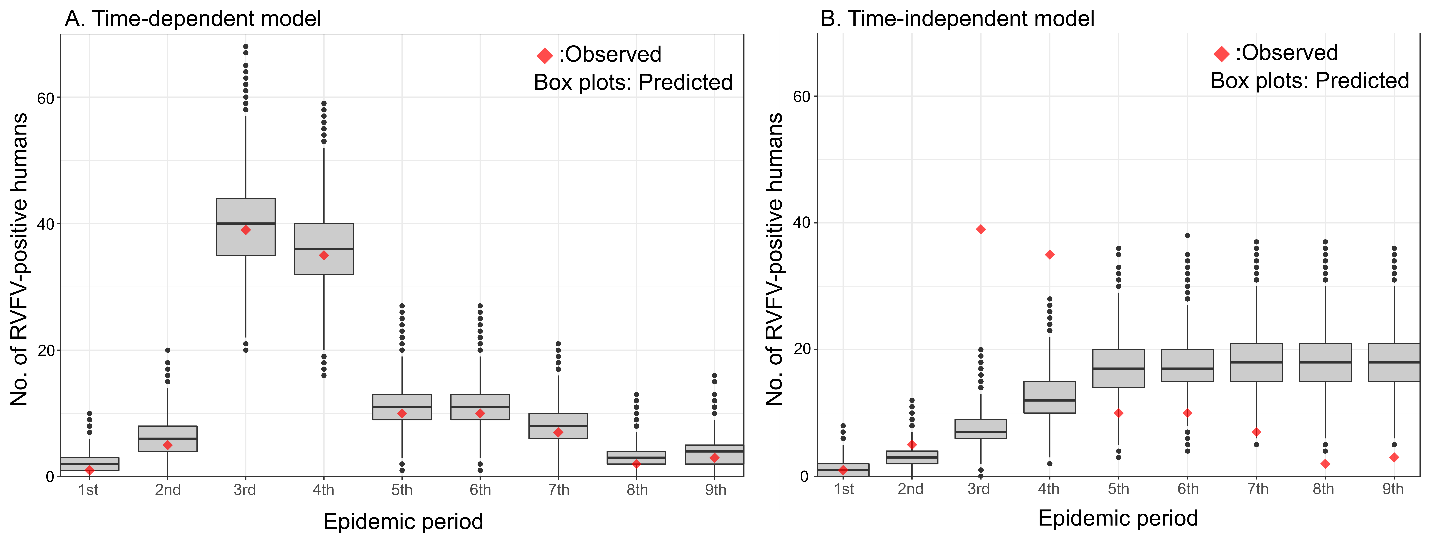
 Fig H.** Posterior predictive check of the **(A)** ‘time-dependent’ and **(B)** ‘time-independent’ models for which the rate of human infection was a function of the distance from livestock-infected communes. Points and box plots represent the observed and predicted numbers of Rift Valley fever virus (RVFV)-positive human cases in each epidemic period, respectively.
